# Oral health and dental care challenges in scleroderma—perspectives of dentists, rheumatologists and patients

**DOI:** 10.1093/rap/rkae121

**Published:** 2024-10-03

**Authors:** Tyler J Mills, Elizabeth Price, Vishal R Aggarwal, Francesco Del Galdo, Liz Walker

**Affiliations:** Faculty of Health Sciences, University of Hull, Hull, UK; Faculty of Health Sciences, University of Hull, Hull, UK; School of Dentistry, University of Leeds, Leeds, UK; Leeds Institute of Rheumatic and Musculoskeletal Medicine and NIHR Biomedical Research Centre, University of Leeds, Leeds, UK; Faculty of Health Sciences, University of Hull, Hull, UK

**Keywords:** scleroderma, systemic sclerosis, oral health, rheumatologists, dentists, quality of life, dental care, United Kingdom

## Abstract

**Objectives:**

The oral healthcare challenges of people living with scleroderma are poorly understood, yet frequently reported. This mixed methods study aimed to investigate oral health and dental care challenges associated with scleroderma from the perspective of dentists, rheumatologists and patients.

**Methods:**

Dentists, rheumatologists and scleroderma patients in the UK completed a survey about their experiences of oral health, dental care and quality of life. People with scleroderma were also interviewed. We conducted descriptive analysis of quantitative data and used thematic analysis to examine qualitative data.

**Results:**

A total of 95.5% of patients reported oral and dental manifestations of scleroderma (ODMS); 57.1% reported high physical impacts, 53.8% high psychological impacts and 47.7% high social impacts. Only 13% of patients were informed of ODMS at diagnosis. No dentist or rheumatologist felt fully confident in managing ODMS. The most frequent suggestion for improvement among patients and dentists was increased information for dentists. We identified three key themes: significant negative impact on quality of life, barriers to accessing dental care and characteristics of good dental care.

**Conclusion:**

ODMS are prevalent issues, constituting a significant burden on quality of life. Rheumatologists should inform scleroderma patients of ODMS and embed oral health inquiries into annual reviews. Communication between medical and dental practitioners should be encouraged to facilitate early identification and management of ODMS.

Key messages

- Oral and dental manifestations of scleroderma (OMDS) present a significant physical, psychological and social burden, which is rarely acknowledged.
- Scleroderma patients are not routinely informed about ODMS, which is a barrier to early intervention.
- Rheumatologists should provide information to patients on ODMS symptoms and review them on an annual basis.

## Introduction

Scleroderma is characterized by significant mortality and morbidity due to its effects on the internal organs and cardiorespiratory and gastrointestinal functions [1]. Yet the oral and dental manifestations of scleroderma (ODMS), which can occur in up to 80% of patients, generate a significant quality-of-life burden [2]. Manifestations may include microstomia, gum disease, loss of dentition and tooth decay [3]. Secondary SS is also common [4]. Research indicates an ‘awareness and referral gap’ between rheumatologists and dentists, which limits our understanding of the experience and impact of ODMS [5]. Furthermore, significant barriers to achieving dental fitness exist for people living with scleroderma. For example, limited dexterity in the hands and restricted mouth opening can limit patients’ ability to maintain dental hygiene [6–8] while presenting particular challenges for dental treatments [9, 10].

Previous research in the USA has highlighted that although 51% of dentists lacked awareness or clinical knowledge of scleroderma, 95% were interested in learning more [11]. However, no studies have explored the perspectives of UK dentists in relation to ODMS. Additionally, research that has explored how to improve the quality of life of people living with scleroderma has stated that increased attention to oral health and ODMS is a research priority for both patients and professionals [12] and the necessity of increased academic and clinical attention to ODMS has been reiterated by recent reviews and meta-analyses [4, 5, 13–16].

This is the first exploration of ODMS that focuses on the perspectives of people living with scleroderma, rheumatologists and dentists. This mixed methods study aims to explore experiences of ODMS as well as barriers and enablers to dental care to identify ways to improve the quality of life of people living with ODMS.

## Methods

### Study design

An explorative mixed methods study was conducted: three online surveys were completed by primary care dentists, rheumatologists and people living with scleroderma. People living with scleroderma were interviewed concurrently. All patient participants had a confirmed diagnosis of scleroderma. Ethical approval was granted by the University of Hull Faculty of Health Sciences Ethics Committee (FHS446) and the National Health Service (NHS) Health Research Authority (15/NE/0211). All findings were reviewed by a patient and public involvement (PPI) group of people living with scleroderma recruited through the National Institute for Health Research (NIHR) Leeds Biomedical Research Centre Patient and Public Involvement and Engagement (PPIE) Group.

### Recruitment

The principal method of patient recruitment was through approaching patients already enrolled in the Stratification for Risk of Progressions in Scleroderma study (15/NE/0211, University of Leeds) [17]. Additionally, a call for patient participants was posted on social media by Scleroderma and Raynaud’s UK (SRUK). Rheumatologists were recruited through a call for participants in the UK Scleroderma Study Group. Dentists were recruited from the British Dental Association and the College of Dentistry, and the call for participants was endorsed by the Oral Health Foundation. Surveys were anonymous and hosted on Jisc [18]. All participants were provided with an information sheet outlining the purpose of the study, what the research involved, considerations of data protection and confidentiality and contact details.

At the end of the survey, patient participants were asked if they wished to take part in a follow-up interview. SRUK shared another round of advertisements for potential participants who did not wish to complete the survey but wanted a one-on-one interview. All potential participants were provided with an information sheet that outlined confidentiality, the purpose of the interview and what would happen to their data. Informed consent was provided by signing an online form or recording a verbal agreement to the informed consent questions.

### Data collection

The patient survey had 158 respondents and ran between June and September 2022. The clinician surveys ran between June and October 2022 and included 25 primary care dentists and 26 rheumatologists. The patient survey included 28 questions: 4 on demographics, 12 on ODMS symptoms and impact and 12 on experiences of health and dental care for ODMS (see Supplementary Table S1, available at *Rheumatology Advances in Practice* online). The dentist survey included 16 questions: 4 on demographics, 4 on awareness and clinical experience with ODMS, 5 on referrals and 3 on confidence and support (see Supplementary Table S2, available at *Rheumatology Advances in Practice* online). The rheumatologist survey included 20 questions: 4 on demographics, 4 on clinical experience with scleroderma, 3 on confidence and experience with ODMS and 7 on referrals (see Supplementary Table S3, available at *Rheumatology Advances in Practice* online).

Interviews with 13 scleroderma patients were conducted between July and December 2022 and lasted up to 120 min. The interviews were conducted online, through Zoom or Microsoft Teams (participant preference) by T.J.M. and were audio-recorded. A semi-structured interview schedule was used (see Supplementary Table S4, available at *Rheumatology Advances in Practice* online). The audio was transcribed verbatim.

### Data analysis

Descriptive analysis of quantitative data was undertaken using SPSS Version 29 (IBM, Armonk, NY, USA) and the qualitative survey data and interview data were analysed separately by T.J.M. using thematic analysis [19]. This involved an inductive and flexible coding process through immersion in the data; coding structure and themes were reviewed by L.W. and E.P. to ensure interrater reliability. The number of participants who contributed to the themes and codes from the analysis of free-text survey responses was noted and was used as a descriptive quantitative measure (e.g. number of patients who experience oral pain) to enhance the qualitative analysis (e.g. the lived experience and impact of oral pain). Supplementary Table S5, available at *Rheumatology Advances in Practice* online, is an illustration of the integrated quantitative and qualitative analyses. A final stage of analysis involved combining all sources of survey and interview data; the resulting themes were discussed and reviewed by all authors. Results were presented to the PPI group over four online video-call sessions. This enabled co-production of awareness-raising leaflets and videos for dentists and patients.

## Results

### Survey: patients

A total of 158 scleroderma patients from all UK nations completed the survey. The most frequent age category was 61–70 years and 97% identified as female (Table 1).

**Table 1.** Demographic characteristics of survey (dentists, rheumatologists, patients) and interview participants

Characteristics	Survey			Interview participants
	Dentists	Rheumatologists	Patients	
*n*	26	25	158	13
Age (years), mode	31–40	41–50	61–70	≥50
Country (*n*)				
England	24	25	130	13
Wales	1	–	8	–
Scotland	–	1	15	–
N. Ireland	–	–	5	–
Gender (*n*)				
Female	6	13	153	12
Male	18	13	5	1
Type of scleroderma (*n*)				
Localized	–	–	4	–
Limited	–	–	86	2
Diffuse	–	–	43	11
Not sure	–	–	25	–
Decade of diagnosis (*n*)				
1980s	–	–	4	2
1990s	–	–	16	1
2000s	–	–	32	2
2010s	–	–	70	5
2020–2022	–	–	26	3

**Figure 1.**
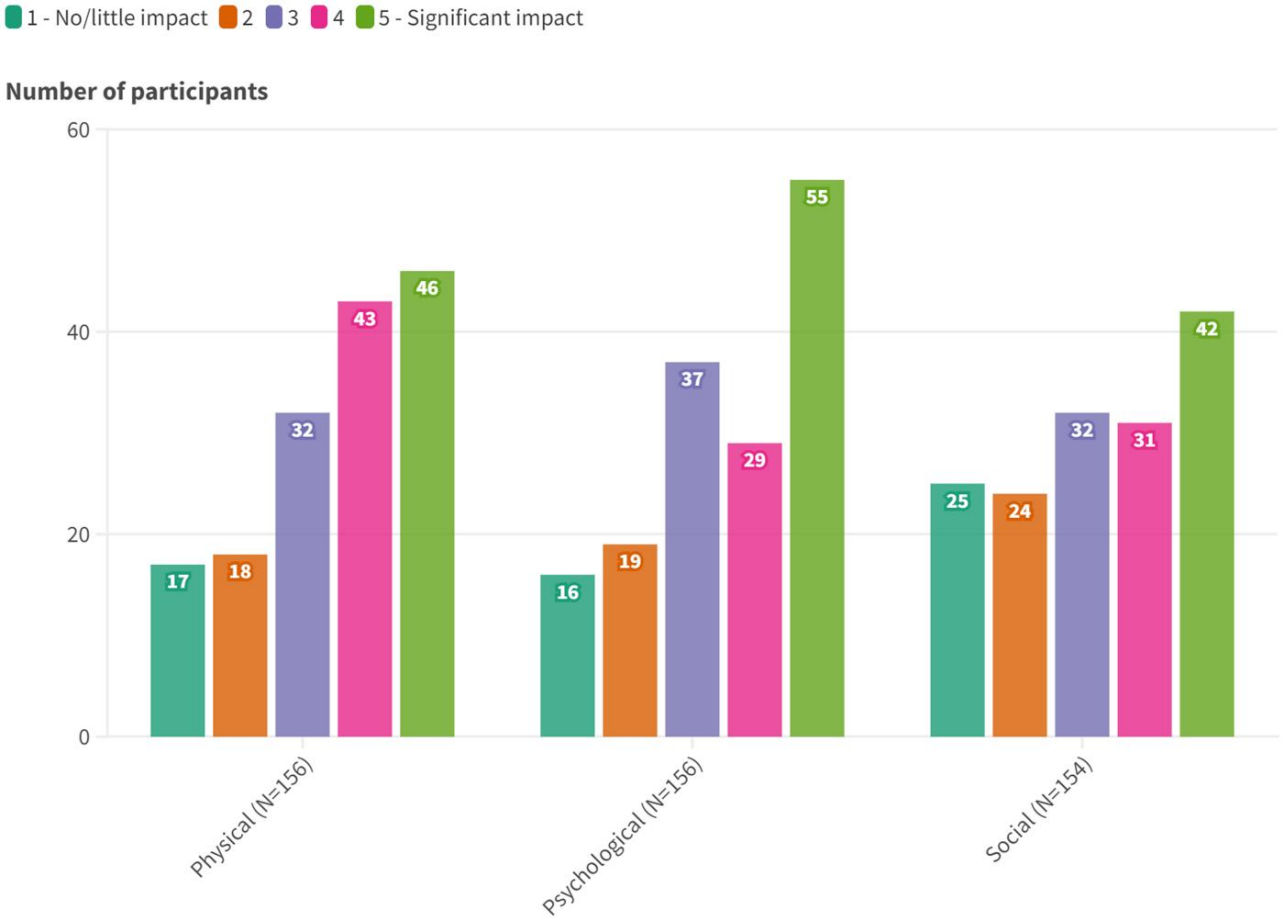
Bar charts of the physical, psychological and social impacts of ODMS

A total of 95.5% (*n* = 150) of participants reported problems with their mouth/teeth (Table 2). Participants’ free-text responses demonstrated a wide range of issues and related impacts (Supplementary Table S5, available at *Rheumatology Advances in Practice* online). Participants rated the physical (*n* = 156), psychological (*n* = 156) and social (*n* = 154) impacts of living with ODMS (Fig. 1) using a 5-point Likert scale ranging from 1 (no/little impact) to 5 (significant impact). For each category, the most frequently selected response was ‘significant impact’. Looking only at the highest impact categories [4 (moderate impact) and 5 (significant impact)], 57.1% (*n* = 89) of participants reported a high physical impact, 53.9% (*n* = 84) of participants reported a high psychological impact and 47.7% (*n* = 73) of participants reported a high social impact.

**Table 2.** Patient survey results

Results	*n*
Problems with mouth/teeth (*n* = 157)	
Yes	150
No	7
Informed of ODMS at point of diagnosis (*n* = 157)	
Yes	20
No	137
Had any clinician suggested oral/dental problems relate to scleroderma? (*n* = 151)	
Yes	83
No	68
Private/NHS dental care (*n* = 158)	
No dentist	8
Private	57
NHS	93
Dentist aware of diagnosis (*n* = 151)	
Yes	139
No	12
Suggestions for improvements (multiple choice) (*n* = 156])	
Greater awareness from dentists	122
Access to specialist dental care	120
Greater awareness from rheumatologists	106
Access to more affordable dentistry	101
Information (for patients) about how to manage orodental problems	97
Access to early information	82
Access to basic dental care	53
Confidence in dentist (*n* = 155)	
Very uncertain	54
Somewhat uncertain	24
Neither uncertain nor confident	46
Somewhat confident	15
Fully confident	16
Confidence in rheumatologist (*n* = 155)	
Very uncertain	47
Somewhat uncertain	48
Neither uncertain nor confident	36
Somewhat confident	10
Fully confident	14

Only 13% (*n* = 20) were informed at diagnosis about the potential orodental manifestations of scleroderma; 10 of these were informed only about microstomia or tightening of the facial skin (Table 2). A total of 55% (*n* = 83) reported that a dentist or rheumatologist had since suggested that their oral/dental problems may be related to scleroderma. A total of 95% (*n* = 150) had a dentist; of these, 62% (*n* = 93) received NHS dental care.

Participants rated their level of confidence in their rheumatologist and dentist regarding the management of ODMS on a 5-point Likert scale ranging from 1 (very uncertain) to 5 (fully confident) (Table 2). The most frequently selected response regarding confidence in the dentist was 1 (very uncertain; *n* = 54), while the most frequently selected response regarding confidence in the rheumatologist was 2 (somewhat uncertain; *n* = 48). Looking only at responses that indicate uncertainty [1 (very uncertain) and 2 (somewhat uncertain)], 50.3% (*n* = 78) of participants felt uncertain about their dentist, whereas 61.2% (*n* = 95) of participants felt uncertain about their rheumatologist with regard to managing ODMS.

Participants chose from a multiple-choice list regarding what might help them to manage ODMS (Table 2). The most frequently selected suggestion was for greater awareness among dentists (*n* = 122), closely followed by access to specialist dental care (*n* = 120). Access to basic dental care was the least selected option (*n* = 53); 34.2% of participants reported they did not have access to basic dental care.

### Survey: primary care dentists

A total of 25 primary care dentists from England and Wales completed the survey; 68% (*n* = 17) reported that they were aware of ODMS (Table 3) and 44% (*n* = 11) of dentists had clinical experience with scleroderma. Limited access to the mouth was noted as a difficulty in relation to patients’ ability to maintain dental hygiene and dentists’ ability to provide treatment. Four dentists had referred patients to rheumatology with suspected scleroderma and the diagnosis was confirmed in two of these cases.

**Table 3.** Primary care dentist survey results

Results	*n*	Text response examples
Familiarity with ODMS (*n* = 25)		
Yes	17	
No	7	
Not aware of scleroderma	1	
Clinical experience with ODMS (*n* = 24)		
Yes	11	‘Difficulty with oral hygiene due to limited mouth openings and limited manual dexterity and limited mouth opening making dental treatment difficult’.
No	9	
Not sure	4	
Referral to rheumatology (*n* = 25)		
Yes	4	‘Dry mouth, tight skin around the mouth and face, blue/white fingers. Difficult to swallow…Confirmed for CREST’.
No	12	‘No—patient died’.
N/A	9	‘They had been seeing a rheumatologist for years before I met them’.
Referral from rheumatology (*n* = 18)		
Yes	5	‘Yes, difficulty accessing dental treatment due to medical history, sore/painful mouth’.
No	13	
Referral to other dental services (*n* = 21)		
Yes	2^a^	
No	19	
Suggested improvements (*n* = 16)		
More information for dentists	5	
Specialist support/point of contact for advice	3	
More awareness about scleroderma in dentistry	2	
More information for patients (leaflet)	2	
Emphasis on prevention and early intervention	2	
Improved access to appropriate dental services and easier referrals	2	
Confidence managing ODMS (*n* = 24)		
Very uncertain	9	
Somewhat uncertain	2	
Neither uncertain nor confident	5	
Somewhat confident	8	
Fully confident	0	

a Oral medicine (*n* = 1) and restorative dentistry (*n* = 1).

Participants rated their level of confidence in managing ODMS on a Likert scale ranging from 1 (very uncertain) to 5 (fully confident). No primary care dentists felt fully confident in managing orodental problems associated with scleroderma. The most frequently selected response was ‘very uncertain’ [36% (*n* = 9)]. Of 25 primary care dentists, 16 provided suggestions of improvements to help them support patients with scleroderma. The most frequently suggested (*n* = 5) was more information for dentists about scleroderma, such as continuing professional development courses or access to specialist advice.

### Survey: rheumatologists

A total of 26 rheumatologists from England and Scotland completed the survey. All had previously worked with scleroderma patients and most had extensive clinical experience, as 69% (*n* = 18) had worked with >50 scleroderma patients (Table 4). All routinely provided disease-specific information to patients at the point of diagnosis, but only one rheumatologist reported routinely providing information on ODMS.

**Table 4.** Rheumatologist survey results

	*n*	Text response examples
Number of patients and presenting issues (*n* = 26)		
1–10	1	‘What’s going to happen to my skin?’
11–20	2	‘Fatigue, Raynaud’s, joint pain/contractures’
21–30	1	‘Medication side effects’
31–40	3	‘Raynaud’s care’
41–50	1	‘Depends very much on the distribution of and presentation of the disease’
≥51	18	‘It’s a huge range. Raynaud’s, fear of the future, skin, GI, lung and sicca related. Medication related’.
Typical post-diagnosis referrals (*n* = 26)		
Respiratory	16	
Occupational therapy	12	
Cardiology	10	
Physiotherapy	7	
Gastro	6	
Dental hospital/oral medicine	5	‘Specialist dental clinic referral IF not able to get good dental care. Try artificial saliva spray and pastilles but open about limitations and discuss water availability’.
Dermatology	4	
Psychological services	2	
Ophthalmology	2	
Neurology	2	
Podiatry	1	
Estimated percentage of patients with orodental concerns (*n* = 26)		
None	3	
<25%	14	‘This has become a particular problem since the NHS dental system has been so difficult to access’
25–50%	8	‘Very much so. Mostly related to oral dryness and reflux, but the oral aperture is an issue and dentists struggle so there are often more complex needs’
70%	1	‘I now specifically ask at least on an annual basis!’
Referral to dental services (*n* = 26)		
Yes^a^	18	‘They were not overly helpful I must say, and I have referred on to a more national service’ ‘I have referred to the [regional] Dental Hospital but they are now starting to reject non-complex referrals’
No	8	
Referral from dental services (*n* = 26)		
Yes	2	
No	24	
Confidence managing ODMS (*n* = 26)		
Very uncertain	4	
Somewhat uncertain	8	
Neither uncertain nor confident	8	
Somewhat confident	6	
Fully confident	0	

a Dental services included primary care dentistry (*n* = 1), secondary care dental hospital (*n* = 7), special care dentistry (*n* = 2), oral surgery (*n* = 3) and oral medicine (*n* = 4).

A total of 88% (*n* = 23) confirmed they had patients who raised orodental concerns and, of these, 61% (*n* = 14) indicated that <25% of their patients mention ODMS. A total of 69% (*n* = 18) indicated they had referred patients to dental services, while only 2 had received a referral from a dentist. Participants rated their own level of confidence in managing ODMS on a Likert scale ranging from 1 (very uncertain) to 5 (fully confident). No rheumatologist in this survey felt fully confident in managing ODMS. The most frequently selected responses were 2 (somewhat uncertain) [31% (*n* = 8)] and 3 (neither uncertain nor confident) [31% (*n* = 8)].

### Thematic analysis: patient survey and interviews

A total of 13 people with scleroderma were interviewed (Table 1); 11 were diagnosed with diffuse scleroderma and 2 with limited scleroderma and the time since diagnosis ranged from 1 to 33 years (see Supplementary Table S6, available at *Rheumatology Advances in Practice* online). Three main themes emerged from the thematic analysis of both open-text comments in patient surveys (*n* = 158) and interviews (*n* = 13): the significant negative impact of ODMS on quality of life, barriers to accessing dental care and characteristics of good dental care. See Table 5 for themes, subthemes and example quotes from the survey and interviews.

**Table 5.** Patient survey and interview thematic analysis: themes and subthemes

Themes	Subthemes	Example quotes (survey)	Example quote (interviews)
Significant negative impact of ODMS on quality of life	Physical	Food packs into the holes in my mouth creating pain. P025 Mouth is smaller, lips thinner, lines around mouth. P048 As we struggle with fatigue we often opt for easy foods and ready meals. When having to consider whether ‘do I have the strength to chew’ or ‘do I have the strength to brush my teeth’, this impacts what we eat further. P029	I’ve lost all my teeth through it; my jawbone has splintered; so, the bone works its way out through the gum at times. They’ve shrunk through the scleroderma and the Sjogren’s because obviously I’ve got no saliva glands…I suppose my teeth, they started to take them out probably about 2014 and, to be honest, a couple of them just fell out one day. IP01
	Psychological	I can see the changes when I look at myself, I feel ugly and deformed on some days. P132 I worry about losing my teeth all the time. P060 [ODMS are] just another reminder that this disease is incurable and destroys every part of our being and who we were before it hit us. P007	You get terrified because it just makes you more different, and people don’t want to be different…you don’t want to lose the ability to vocalise and to talk and, you know, this is your expression of yourself but also, you don’t want to be in pain and you don’t want to have really bad teeth. IP04
	Social	I don’t go out and socialise as I feel people are staring at my teeth. P057 I have lost friends because they don’t understand. P120 I avoid eating out with family and friends; it takes ages to clean my teeth and I feel awkward. P062 I don’t like speaking to anybody because I cannot pronounce words correctly. P153	I don’t go for a meal with friends and that sort of thing in the evening…I don’t have quite so much feeling in my lips as I used to, that can make eating quite hard; so I tend to think well I don’t know if I really want to go and (laughs) eat in front of other people, so sometimes I just avoid that as well. So, it has affected what I do on a social front. IP10
Barriers to accessing dental care	Lack of information and awareness	I worry about going to the dentist as they don’t understand I can’t open my mouth wide. P143 I don’t think my mouth and teeth are taken as seriously as the other tests and treatments I receive. P024 Feel like dentist judges me, blames me even though I clean my teeth! P035 I feel my dental practice ignores my medical condition. P137 Not discussed by rheumatology consultant all. Felt rheumatology not interested in mouth complications. P010 Feel strongly that rheumatology doesn’t consider oral health as important as other symptoms. P011	I found out that apparently [the diagnosis] was 11 years ago but no-one, no-one told me, and so I’ve been muddling along for all this time with not knowing, so that wasn’t good. IP07 Nothing was ever said about oral matters or dental matters. IP03 You clean your teeth and look after them and all of a sudden I’m losing ’em, and it’s like why? what’s going on? I didn’t find enough information at the time on what was happening; so for me, I would have liked to have understood a bit more about oral care. IP01
	Inconsistent and difficult access to dental care	In my experience, NHS dentists either did not know how or have funding for my dental issues, which is why I pay monthly to go privately. P068 Can’t afford to have private treatment at the moment even though I need dental work. P110 Despite my requests to rheumatologist for past 3 years to get referral to specialist dental centre it took virtually all my front teeth to be lost to get a referral to my local hospital dental restorative unit. P149 NHS dentists constantly changing so no continuity. P095	Financial drain is really important…the thing is that you have to try a lot of things, so you pay a lot of money to try different things, but also obviously most of that, because it’s special and, as a disability aid or anything like that, it’s just twice the price, right?…like it’s easy to say ‘oh buy yourself an electric toothbrush’, but that might be another £70 right, that you just do not have because you spent so much other money. IP04
Characteristics of good dental care	Information provision, knowledge, and compassion	More understanding by dentist. P072 It would help if both the medical and dentists knew what this disease is and its effects. P041 My dentist was very good and did X-rays and researched scleroderma/SS when I was diagnosed. P018 Make dentist more aware of the condition and have training on treatments to help patients. P054 Education of dentists is vital. They may cover the condition in their degree courses, but not enough to be able to deal properly with our problems. P076 Leaflets from rheumatology to give to our dentists with necessary info and what to look out for. P018 The experience with my implant showed me that even if you think scleroderma makes dental work impossible, an understanding dentist makes a huge difference. P062	It would be good if you could see someone who actually knew all about it before you had to say anything about it…rather than having to go and seek all this information out; it would be good if there was a support package, even if it was just a better leaflet system, or just, access to advice that you don’t have to search out yourself. IP10 Something for the dentist, something like a pamphlet or leaflet where it says, you know, these are often the comorbidities, this is what it means, you know, with scleroderma these are the common problems, you know, like receding gums, bleeding gums, shortened tongue…like all these things that’s like, that might not be immediately apparent. IP04
	Cooperation and coordination: early access to dental care	There is currently no link between my dentist for ongoing care and my rheumatology team. If this could be offered I’m sure there would be advantages for all parties. P099 I am hoping for a referral to a scleroderma-aware dentist. P103 I’ve had several symptoms of scleroderma for years and having so many professionals looking at symptoms individually has hindered a diagnosis. P068 I think we should be offered free treatment and any treatment we need by the dentist free due to the fact that we are directly affected by this chronic condition with our teeth and gums. P060	Earlier access, definitely; I think if a patient has got microstomia and they say to their rheumatology consultant, I’m having problems with my teeth, that should be an immediate referral to the hospital dentistry, not just an NHS dentist who gives little acknowledgement to the difficulties that we’re going to be facing down the line. I think they’ve got to think more long-term, definitely, because I think had that been acknowledged 5 years ago for me when these major problems started to occur…and had they been dealt with at that point, teeth filled correctly, it would have been much better, my mouth might be in a better condition. IP02

### Significant negative impact of ODMS on quality of life

#### Physical impact

Participants struggled with a range of ODMS that had a negative physical impact; pain and sensitivity was the most reported, limiting the ability to eat. Additionally, participants highlighted that managing ODMS was a time-consuming and emotionally draining task, further complicated by fatigue and functional limitations. All those interviewed struggled with multiple ODMS and, for some, these had worsened over time.

‘I’ve lost all my teeth through it; my jawbone has splintered; so, the bone works its way out through the gum at times’. — IP01

#### Psychological impact

Living with orodental problems was described as ‘depressing’ and ‘a daily struggle’. Participants felt ‘drained’ by the necessity of daily management of symptoms and dental hygiene. The psychological toll was heavy and vividly clear in descriptions of ODMS as

‘…just another reminder that this disease is incurable and destroys every part of our being and who we were before it hit us’. — P007

The psychosocial impact of ODMS was often seen as a hidden issue:

‘It isn’t just a physical thing, it definitely affects self-confidence and the way you react socially’. — IP04

#### Social impact

Living with ODMS was described as a hidden struggle; participants felt intense embarrassment about their oral health and were often reluctant to share their worries with others, sometimes due to concerns about losing social connections.

‘I don’t tell anyone [how] I feel’. — P062

Avoidance of eating with other people limited opportunities for socializing, and many people tried not to smile, laugh or otherwise show their teeth. Some struggled with functional limitations to speech, and multiple participants described feeling discouraged from sharing meals due to dietary restrictions and intensive oral hygiene maintenance.

### Barriers to accessing dental care

#### Lack of information and awareness

None of the interviewed participants felt they had been provided sufficient information about scleroderma. Of those who did receive information, none were informed about ODMS. The lack of information and awareness of ODMS among medical and dental professionals was highly distressing for participants. Furthermore, when participants attempted to inform their dentist about scleroderma, many did not feel believed or understood and felt afraid of going to the dentist. Those who approached their rheumatologist similarly felt as though the impact of ODMS was not truly acknowledged.

‘I actually walked out [of the dental appointment] because…he just didn’t know what to do, you know. I explained everything and he just looked at me and said, “what does that mean?”’. — IP01

#### Inconsistent and difficult access to dental care

Many participants struggled to access NHS dental care; some could not find an NHS dentist, and when they did, there was no continuity between dentists. Referrals to dental hospitals or specialist dental centres were notoriously difficult to obtain; referrals were often not accepted until the progression of ODMS limited treatment options. Dental care constituted a heavy financial burden; many participants required extensive (and expensive) dental interventions but were also on a low income or disability benefits.

‘I wasn’t able to get an NHS dentist unfortunately, I had to go private for it, which cost me an arm and a leg and a first born!’ — IP11

Many also highlighted that the fluctuating and multifaceted nature of ODMS necessitated specialist aids and products, which was often a case of trial and error at their own expense.

### Characteristics of good dental care

#### Information provision, knowledge and compassion

Participants were aware that scleroderma is a rare condition and emphasized that their positive experiences were not dependent on the dentist having detailed prior knowledge. Rather, it was important that clinicians listen to the lived experience of patients with compassion and do their own research into ODMS. One participant (IP11) described how a dentist had removed all his teeth without understanding the progressive changes to his mouth that scleroderma could, and would, cause. This left him unable to wear the dentures he was provided with, noting that this may not have happened if both he and his dentist were made aware of ODMS. Multiple participants stated that their oral and dental care would have been improved by the provision of reliable information at the point of diagnosis.

‘If I go right back to the beginning it would have helped, I suppose, if I’d been made more aware…Just so that dentists in general are aware of scleroderma and actually what can happen inside a person’s face if they have to have their teeth out and have dentures put in’. — IP11

#### Cooperation and coordination: early access to dental care

Earlier access to information and access to specialist dental care was seen as a priority for participants in both the survey and interviews. Multiple participants felt that any scleroderma patient who has ODMS should receive a referral to a dental specialist, ideally at low-cost through the NHS, before progression of scleroderma limits access to the mouth. Some felt their symptoms would have been recognized as scleroderma earlier had their rheumatologist been in contact with their dentist.

‘I’ve had several symptoms of scleroderma for years and having so many professionals looking at symptoms individually has hindered a diagnosis’. — P068

Participants also suggested that increased communication between rheumatologists and dentists would improve their experience.

## Discussion

This is the first research to triangulate the views of patients, rheumatologists and dentists to explore oral health and dental care in people with scleroderma with the aim of identifying barriers and exploring potential solutions to improve quality of life. This is also the first UK study to explore dentist perspectives of ODMS. The results demonstrate that ODMS are prevalent among patients and that they confer a substantial physical, psychological and social burden. There is currently little communication between clinicians, presenting a barrier to early intervention, and neither rheumatologists nor dentists felt fully confident in managing ODMS. The quantitative and qualitative findings were integrated across patients, rheumatologists and dentists to provide a unique insight into the difficulties ODMS present and potential avenues for mitigating these.

Despite the high prevalence of ODMS, as noted among both patients and rheumatologists, and the substantial negative impact on quality of life for patients, our findings indicate that rheumatologists did not routinely provide information or inquire about ODMS. The consequences of delaying information provision, and therefore delaying early identification and management of ODMS, are widespread. First, patients are not aware of what help is available, a significant barrier to achieving dental fitness before scleroderma limits access to the mouth [3, 4, 10, 14]. Second, the lack of information is detrimental to patients’ well-being. Numerous patients were unaware that scleroderma can manifest in the mouth. Thus when they experienced ODMS, they did not have the information necessary to seek appropriate care. Furthermore, patients reported feeling ignored or invalidated when raising oral health concerns; a common perspective was that dentists did not understand ODMS and that rheumatologists did not care. Greater awareness of ODMS from dentists [78% (*n* = 122)] and rheumatologists [67.9% (*n* = 106)] were clear patient priorities, supporting previous research illustrating similar difficulties faced by patients [6, 11, 12]. We therefore recommend that rheumatologists should inform all scleroderma patients about ODMS and provide information very early in their patient journey and should inquire about their oral health annually. An outcome of this research was the co-production of awareness-raising material about ODMS, developed in collaboration with patients and clinicians. This includes animated videos and information cards, endorsed by SRUK and the Oral Health Foundation, which rheumatologists can use to raise awareness of ODMS among patients and dentists [20, 21].

Our study revealed that no clinician felt fully confident in managing ODMS, however, many offered suggestions for improvements, including more information and training. These results confirm the findings of previous studies that indicated dentists lacked knowledge of scleroderma, yet were interested in learning more [11]. Crucially, across patient and clinician surveys, all indicated that the early identification and management of ODMS is vital to preserve dentition and achieve dental fitness. Previous studies have demonstrated that dentists are uniquely placed to facilitate early diagnosis of scleroderma through identification of pathognomic ODMS, such as widening of the periodontal ligament space and mandibular bone resorption [4, 5, 22]. Our research revealed that multiple patients believed their symptoms could have been diagnosed as scleroderma earlier or they could have received more appropriate dental care if their rheumatologist and dentist had been in communication. Patients’ narratives of diagnostic delay are supported by a recent analysis of the epidemiology of scleroderma in the UK that indicated a significant delay between identification of RP and diagnosis of scleroderma [23] despite evidence indicating that RP is a useful indicator to facilitate early diagnosis [24]. However, these studies did not include ODMS in their analyses. Additionally, studies that explored the success rates of dental surgery in scleroderma patients all emphasize the need for rheumatologists and dentists to collaborate on treatment planning and monitoring [9, 14, 25, 26]. The consequences of this delay are evidenced in our research and in extant literature [6, 9, 14], thus it is critical that rheumatologists and dentists communicate to share knowledge and coordinate the management of ODMS early in the disease process.

It is clear from both quantitative and qualitative patient data that ODMS present a significant, often unacknowledged, burden on quality of life. Patients reported that the physical, psychological and social impacts of ODMS are frequently experienced and to such an extent that their lives are severely restricted. There was a sense of hopelessness and despair, partly arising from a lack of information and options for support. Negative dental experiences affected participants’ confidence in clinicians. This is important to acknowledge, because maintenance of oral hygiene routines are influenced by emotional well-being [6], and previous research has demonstrated that having an unmet dental need and expressing a lack of trust in one’s dentist are significant predictors of poor oral health [27]. Therefore, it is advantageous to both clinicians and patients to build a trusting working relationship in pursuit of positive outcomes. Our results, in line with extant literature on relationships between dentists and patients with chronic health conditions, highlight factors that facilitate trust between scleroderma patients and their dentists [5, 6, 27–29]. These include the dental practitioner learning about the medical condition(s) of the patient through communication and self-education, a compassionate understanding of how restricted mobility and emotional distress may be a barrier to oral health and adapting treatment plans according to dental needs and biopsychosocial barriers experienced by the patient. To this end, we co-produced an information video and a printable folding wallet card to help patients inform dentists of the key issues related to ODMS and their dental care. These awareness-raising materials are endorsed by the Oral Health Foundation and can be accessed on the SRUK website [20] or via OSF [21].

This is the first research in the UK to examine ODMS from the perspective of patients, dentists and rheumatologists. The mixed methods approach allowed us to comprehensively explore the topic while centralizing lived experiences. Similar research in the USA [11] and Canada [12] has benefitted from recruiting participants from extensive, specific networks; purposive sampling from networks of clinicians could provide a more representative sample in future studies. Furthermore, although all patients had a confirmed diagnosis of scleroderma, some were not aware of the specific type and others were unsure of their comorbid diagnoses, therefore it is difficult to delineate how these factors may have influenced patient experiences.

This article illustrates the challenges ODMS present to scleroderma patients and clinicians. The lack of awareness of ODMS and lack of information provision to patients is a barrier to effective dental care, which could be rectified by rheumatologists embedding routine inquiry regarding ODMS into clinical practice. To enable appropriate and timely dental care, access to NHS dentistry should be prioritized for scleroderma patients. Additionally, we suggest that increased communication and multidisciplinary collaboration between dental and medical professionals is essential to enable early identification and management of ODMS. To facilitate enhanced communication and knowledge of ODMS, we encourage dentists to learn more about the oral health experiences of scleroderma patients; our short animated video was designed in collaboration with scleroderma patients to raise awareness among dentists [20]. It is clear that ODMS present a substantial, yet rarely acknowledged, burden to quality of life for people with scleroderma. Patient narratives revealed the hidden battle faced in their pursuit of oral health, and it is imperative that future research determines how best to support patients. In doing so, researchers and clinicians, together with patients, have the potential to pave the way toward more ethical, empowering and effective oral healthcare.

## Supplementary Material

rkae121_Supplementary_Data

## Data Availability

The data underlying this article cannot be publicly shared due to potentially sensitive patient information. SRUK ‘The Mouth in Scleroderma’ resources are available via https://www.sruk.co.uk/about-us/news/scleroderma-mouth-tools-help-dentists-better-under and archived via OSF (https://doi.org/10.17605/OSF.IO/TU8MV).
