# Supplementary material for: Oral health and dental care challenges in scleroderma—perspectives of dentists, rheumatologists and patients
Source: Rheumatol Adv Pract. 2024 Oct 3;8(4):rkae121. doi: 10.1093/rap/rkae121 (PMC11530228; doi:10.1093/rap/rkae121)
Supplement: rkae121_Supplementary_Data [file rkae121_supplementary_data.docx]

# Supplementary Files

## Supplementary Table S1: Patient survey structure

| **Demographic** | 1. What age are you? [categories, under 20, 20-30, … 100+] |
| --- | --- |
|  | 1. What is your gender? [Free text] |
|  | 1. Where do you live? [categories: UK geographic regions] |
|  | 1. Which of the following best describes your ethnic group? [categories from UK census] |
| **Scleroderma and ODMS** | 1. Could you tell us when you were diagnosed with scleroderma and who made the diagnosis? [Free text] |
|  | 1. What type of scleroderma do you have? [categories: diffuse, limited/CREST, localised, not sure] |
|  | 1. Do you have problems with your mouth and teeth? [yes \| no] |
|  | 1. What sort of problems do you have? [Free text] |
|  | 1. Has anyone, such as your rheumatologist or dentist, suggested that these problems may be related to scleroderma? [Free text] |
|  | 1. How much do your mouth and teeth problems impact you physically? [5-point Likert] |
|  | 1. Could you tell us more about the physical impact? [Free text] |
|  | 1. How much do your mouth and teeth problems impact you psychologically? [5-point Likert] |
|  | 1. Could you tell us more about the psychological impact? [Free text] |
|  | 1. How much do your mouth and teeth problems impact you socially? [5-point Likert] |
|  | 1. Could you tell us more about the social impact? [Free text] |
|  | 1. Please use this space to tell us anything else you feel is important for us to know about the impact of your mouth and teeth problems. [Free text] |
| **Medical and dental care for ODMS** | 1. When you were first diagnosed with scleroderma, did your rheumatologist or other professional tell you about the problems scleroderma can cause with the mouth/teeth? [yes \| no] |
|  | 1. If you answered yes, what were you told about the problems scleroderma can cause with the mouth/teeth? [Free text] |
|  | 1. How confident are you that your rheumatologist can understand and support you with any mouth/ teeth problems you have that may be related to scleroderma? [5-point Likert] |
|  | 1. Do you have a dentist? [yes \| no] |
|  | 1. If you have a dentist, do you see them through the NHS or privately? [NHS \| Private] |
|  | 1. Does your dentist know you have scleroderma? [yes \| no] |
|  | 1. How did your dentist find out you have scleroderma? [Free text] |
|  | 1. How confident are you that your dentist can understand and support you with any mouth/ teeth problems you have that may be related to scleroderma? [5-point Likert] |
|  | 1. Have you faced any barriers accessing dental treatment and/or support for your scleroderma related mouth/dental problems? If so, could you tell us about them here. [Free text] |
|  | 1. What sorts of things do you think might help you to better manage the oral/dental problems associated with scleroderma? [multi answer, 7 options] |
|  | 1. Are there any other things you think might help? [Free text] |
|  | 1. Is there anything else you'd like to tell us about your experiences of the dental/mouth related issues that can be related to scleroderma? [Free text] |

## Supplementary Table S2: Dentist Survey Structure

| **Demographic** | 1. What age are you? [categories, under 20, 20-30, … 100+] |
| --- | --- |
|  | 1. What is your gender? [Free text] |
|  | 1. Which geographical area do you work in? [categories: geographic regions] |
|  | 1. What percentage of your work is undertaken privately? [categories, 0-10, 11-20 … 100%] |
| **Awareness and clinical experience with ODMS** | 1. Are you familiar with the oral and dental problems associated with scleroderma? [yes \| no \| not aware of the condition] |
|  | 1. Have you treated patients with scleroderma? [yes \| no \| not sure] |
|  | 1. If so, could you estimate how many? [Free text] |
|  | 1. Could you briefly tell us what problems your patients have had? [Free text] |
| **Referrals** | 1. Have you referred any of your patients to a rheumatologist or other specialist because you thought they may have scleroderma? If so, can you recall what the presenting symptoms were? [Free text] |
|  | 1. Do you recall the outcome of the referral? [Free text] |
|  | 1. Have you referred patients with scleroderma to other dental services? [yes \| no] |
|  | 1. If so, do you recall which one(s)? [categories: special care, secondary care, oral surgery, oral medicine, restorative dentistry, other] |
|  | 1. Have you ever received a referral from a rheumatologist or other specialist due to dental/oral problems associated with scleroderma? If so, can you recall what the presenting symptoms were? [Free text] |
| **Confidence and support** | 1. How would you rate your confidence in identifying the oral and dental problems associated with scleroderma? [5-point Likert] |
|  | 1. Is there anything you feel might help you to support people living with scleroderma? [Free text] |
|  | 1. Is there anything else you'd like to tell us about your experiences of working with people living with scleroderma? [Free text] |

## Supplementary Table S3: Rheumatologist survey structure

| **Demographic** | 1. What age are you? [categories, under 20, 20-30, … 100+] |
| --- | --- |
|  | 1. What is your gender? [Free text] |
|  | 1. Which geographical area do you work in? [categories: geographic regions] |
|  | 1. Where do you primarily work? [general rheumatology \| specialist scleroderma service \| both] |
| **Clinical experience with scleroderma** | 1. Have you worked with people living with scleroderma? [yes \| no] |
|  | 1. Approximately how many people living with scleroderma have you worked with? [categories: 1-10, 11-20, 21-30, 31-40, 41-50, 50+] |
|  | 1. At the point of diagnosis, what information on scleroderma do you provide for patients? [Free text] |
|  | 1. What are the most common issues patients ask/tell you about in consultations? [Free text] |
|  | 1. What post-diagnosis referrals do you ordinarily make for people living with scleroderma? [Free text] |
| **Confidence and experience with ODMS** | 1. How would you rate your confidence in managing the oral and dental problems associated with scleroderma? [5-point Likert] |
|  | 1. In consultations, have you had experience of people raising the issue of the oral and dental problems associated with living with scleroderma? [Free text] |
|  | 1. If your answer is yes, please could you estimate the percentage of patients who raise the issue of the oral and dental problems associated with living with scleroderma. [Free text] |
|  | 1. When patients do raise oral/dental issues in consultations, what are their principal concerns? [Free text] |
| **Referrals** | 1. What dental services are you aware of that might be helpful to people living with scleroderma (other than primary care dentistry)? [Free text] |
|  | 1. Have you ever received a referral from a primary care dentist for a person who was subsequently diagnosed with scleroderma? [yes \| no] |
|  | 1. If you have received a referral from a primary care dentist for a person who was subsequently diagnosed with scleroderma, can you recall what the presenting symptoms were? [Free text] |
|  | 1. Have you referred patients with scleroderma to dental services? [yes \| no] |
|  | 1. If so, do you recall which one(s)? [categories: special care, secondary care, oral surgery, oral medicine, restorative dentistry, other] |
|  | 1. If you selected Other, please specify: [Free text] |
|  | 1. Do you recall the outcome of the referral? [Free text] |

## Supplementary Table S4: Patient Interview Topic Guide

| **Opening questions** | Can you tell me a bit about yourself? |
| --- | --- |
|  | What does a regular day look like?   - Prompt: hobbies, family, work |
| **Diagnosis** | Can you tell me about when you were first diagnosed with scleroderma?   - When was the diagnosis? - How long did it take to get a diagnosis? - What symptoms did you first notice? - How did you feel at the time (before/after diagnosis)? - What impact did it have? |
|  | What is your condition like currently?   - Has it changed since the diagnosis? - What are your main symptoms and how do they affect you? |
|  | What information about scleroderma were you given when you were first diagnosed?   - Any information about oral and dental symptoms? Or about oral hygiene and dental treatment for people with scleroderma? - Offered support? (occ health, psychologist, physio, referral to specialists) - Was what you received sufficient? |
| **Experiences of ODMS** | Do you experience any problems with your mouth or your teeth because of scleroderma?   - What are the problems? |
|  | Can you tell me about the impact that these problems have had on your life?   - E.g., physically, socially, psychologically, financially - Possible follow up: coping strategies; adjustments to daily living; feelings, thoughts, beliefs about health/illness |
|  | What support or treatment have you received (or are currently receiving) for oral and dental problems?   - Dentistry, rheumatology, social or psychological support, support from family/others with scleroderma? |
| **Healthcare and dental care for scleroderma** | Picture what ideal healthcare and dental care for scleroderma would look like for you and describe it to me.   - What is important to you in healthcare and dental care? Why? - What is not important to you and why? |
|  | How happy are you with the healthcare and dental care you currently receive?   - If happy – what made it a positive experience? - If not happy – what made it a negative experience? How can things be improved? |
|  | Are there any treatments or types of support you would like to receive for scleroderma but haven’t been able to?   - What is preventing you from accessing it? - Has this been discussed with healthcare professionals? |
|  | How do you feel about your dentists’ ability to provide good dental care for you as a person with scleroderma?   - Is there anything that could improve your confidence in your dentist? - Or: what are they doing well? |
| **Closing questions** | If you needed more information or support about living with scleroderma, would you know how to access this?   - If not, direct to info sheet, or other resources specific to the issue |
|  | Is there anything that you would like to add that we haven’t discussed?   - Any questions or concerns? |

## Supplementary Table S5: Physical, Psychological, Social impact – Quotes

|  |  | **N of codes** | **Example Quote** |
| --- | --- | --- | --- |
| **Physical impact [N of participants =138]** | Pain and sensitivity | 48 | “I have to sit or lay down propped up as my head feels like it’s going to explode, when my teeth and jaw bones etc. hurt I can’t do anything”. **P117** |
|  | Microstomia | 29 | “My dentures cause me pain regularly because they quickly become Ill fitting because my mouth seems to be shrinking over time.” **P118** |
|  | Restricted ability to eat and enjoy eating | 65 | *“The mouth ulcers can be very painful and can interfere with eating and drinking when they are bad.”* **P154** |
|  | Sjogren’s/sicca | 26 | “Constant dry mouth, lips stuck to teeth, dry throat” **P010** |
|  | Damaged teeth | 18 | *“Teeth changed e.g. alignment, decay, cracks, breakages.”* **P059** |
|  | Difficulty maintaining dental hygiene | 14 | “*My lack of finger dexterity coupled with my shrinking mouth doesn’t allow me to access my mouth”* **P118** |
|  | Changing facial appearance | 21 | *“My lips are getting thinner and my upper lips is restricted which is very uncomfortable.”* **P132** |
|  | Affects speech | 7 | *“It is affecting my speech an awful lot”* **P153** |
|  | Gum problems (receding, bleeding) | 11 | *“Lots of blood and sore gums when brushing”* **P143** |
|  | Cannot use dental prosthetics | 8 | *“Had all my teeth out now to try to solve this … I have no teeth, my dentures will no longer fit.”* **P052** |
| **Psychological impact**  **[N of participants =132]** | Anxiety about deteriorating health | 25 | “*Worry about the future and how things may progress”* **P026** |
|  | Self-conscious of appearance | 31 | *“I am super conscious about my teeth and the fact that I wear a denture. The fact that my body and skin (as well as facial pigmentation) have significantly changed since my diagnosis, definitely impacts me psychologically.”* **P029** |
|  | Embarrassment | 26 | “Embarrassing, affects confidence and can affect voice as if talk too much mouth gets so dry teeth stick to gums” **P065** |
|  | Depression | 18 | “Looks bad, depression, less social, less confidence, waiting to be told its time for false teeth, micro stomia, look like a smoker, people think I don't take care of my teeth.” **P012** |
|  | Dental anxiety | 16 | “*I worry about my teeth falling out, I worry about going to the dentist as they don’t understand I can’t open my mouth wide.”* **P043** |
| **Social Impact [N of participants =130]** | Financial burden of frequent dental care | 51 | “*Rely on disability benefits = no ability to pay for treatment via NHS or private (no choice/control). I also have to buy special toothpaste which is expensive.”* **P059** |
|  | Avoidance of laughing/smiling | 25 | *“I try not to smile or laugh now and try to bow my head or cover my mouth when I talk. My smile used to be something I was proud of, as my teeth have always been white, and something I took pride in looking after. I try not to socialise at the moment because of my lack of confidence due to teeth loss”* **P105** |
|  | Avoidance of socialising | 39 | *“I don't smile. I don't like to talk to people. I avoid social situations.”* **P025** |
|  | Avoidance of eating with others | 46 | *“I am embarrassed to eat in public. Also, the length of time it takes me to eat. I can't eat some foods that I would like to because of chewing and swallowing problems. I also get embarrassed having to drink everything through a straw to avoid dribbling.”* **P142** |

## Supplementary Table S6: Interview participant pseudonyms and characteristics

| Interview Participant ID | Gender | Age | Type of scleroderma* | Co-morbidities* | Time since diagnosis | married/live with partner/ alone | Employed/  Retired | Private/NHS dentist |
| --- | --- | --- | --- | --- | --- | --- | --- | --- |
| IP01 | female | 50+ | systemic scleroderma | Sjogren’s + lupus | 15 years (2007) | - | retired - medical reasons (2011) | - |
| IP02 | female | 50+ | diffuse scleroderma | Sjogren’s | 10 years (2012) | married (husband) | retired - medical reasons (2021) | private [poor access to NHS dental care] |
| IP03 | female | 50+ | systemic scleroderma | MCTD^a^ [RA^b^ + Lupus] | 1 year (2021) | married (husband) | not currently employed | private [poor access to NHS dental care] |
| IP04 | female | under 40 | systemic scleroderma | Sjogren’s | 1.5 year (2021) | living with partner | Adapted employment (work at home) | private |
| IP05 | female | 50+ | systemic sclerosis | Sjogren’s, MCTD, fibromyalgia | 6 years (2016) [prev. dx CREST, 1988] | married (husband) | retired - medical reasons [volunteers] | private |
| IP06 | female | 60+ | systemic scleroderma | Sjogren’s | 23 years (1999) | married (husband) | employed | private |
| IP07 | female | 60+ | limited scleroderma | Sjogren’s | 1 year (2021) [prev. dx CREST, 2011] | - | self-employed | NHS |
| IP08 | female | 50+ | limited scleroderma | Type-1 diabetes | 5 years (2017) | married (husband) | retired | private |
| IP09 | female | 50+ | systemic scleroderma | Sjogren’s | 33 years (1989) | married (husband) | retired [volunteers] | NHS |
| IP10 | female | 60+ | systemic scleroderma | - | 6 years (2016) | married (husband) | self-employed | private |
| IP11 | male | 60+ | systemic scleroderma | Cardiac problems | 2 years (2020) | living with partner | retired | private [poor access to NHS dent care] |
| IP12 | female | 50+ | systemic scleroderma | - | 22 years (2000) | married (husband) | self-employed | NHS |
| IP13 | female | 50+ | systemic scleroderma | Fibromyalgia + lung problems | 6 years (2016) | lives alone | unemployed - medical reasons | NHS |
| *as described by the participant  ^a^ MCTD = Mixed Connective Tissue Disease  ^b^ RA = Rheumatoid Arthritis | | | | | | | | |
